# Quantification of Symptom Load by a Disease‐Specific Questionnaire HPQ 28 and Analysis of Associated Biochemical Parameters in Patients With Postsurgical Hypoparathyroidism

**DOI:** 10.1002/jbm4.10368

**Published:** 2020-06-05

**Authors:** Deborah Wilde, Lara Wilken, Bettina Stamm, Christina Heppner, Andreas Leha, Martina Blaschke, Christoph Herrmann‐Lingen, Heide Siggelkow

**Affiliations:** ^1^ Clinic of Gastroenterology and Gastrointestinal Oncology University Medical Center Goettingen Goettingen Germany; ^2^ Endokrinologikum Saarbruecken Saarbruecken Germany; ^3^ MVZ Endokrinologikum Goettingen Goettingen Germany; ^4^ Institute for Medical Statistics, University Medical Center Goettingen Goettingen Germany; ^5^ Department for Psychosomatic Medicine and Psychotherapy University Medical Center Goettingen Goettingen Germany

**Keywords:** CALCITRIOL, CALCIUM–PHOSPHATE PRODUCT, HYPOPARATHYROIDISM, QUALITY OF LIFE, QUESTIONNAIRE, SYMPTOM LOAD

## Abstract

In hypoparathyroidism (HypoPT), patients suffer severely from reduced quality of life. The complexity of HypoPT demands a disease‐specific control instrument to characterize symptom load. We employed a newly developed disease‐specific Hypoparathyroid Patient Questionnaire (the HPQ 40/28) to investigate and quantify HypoPT patients' complaints and contributing factors. In this cross‐sectional, two‐center study, patients with postsurgical HypoPT (*n* = 49) were matched for gender and age and compared with patients having undergone thyroid surgery without HypoPT (*n* = 39) and patients with primary hyperparathyroidism (*n* = 35). The HPQ 40/28 was completed when patients visited the respective center. Clinical background information, blood tests, and current medication were documented by the physician. Serum calcium lay within the reference range in 87% of HypoPT patients, serum phosphate in 95.7%, and calcium–phosphate product (CPP) in 100%. HPQ 40/28 scores for the scales “pain and cramps” (PaC), “neurovegetative symptoms” (NVS), “numbness or tingling,” and “heart palpitations” were significantly elevated in comparison with control groups. Correlations between complaints and laboratory parameters could be demonstrated in the HypoPT group, with serum calcium correlating with NVS (*r* = 0.309, *p* < 0.05) and serum phosphate with loss of vitality (*r* = 0.349, *p* < 0.05). CPP was the main contributor to symptom load with an influence on PaC (*r* = 0.295, *p* < 0.05), loss of vitality (*r* = 0.498, *p* < 0.001), numbness or tingling (*r* = 0.328, *p* < 0.05), and memory problems (*r* = 0.296, *p* < 0.05). In conclusion, the newly developed HPQ 40/28 successfully identified and quantified symptoms typical in HypoPT patients. Using the HPQ 40/28, the CPP was identified as the predominant factor in the severity of complaints in HypoPT. © 2020 The Authors. *JBMR Plus* published by Wiley Periodicals, Inc. on behalf of American Society for Bone and Mineral Research.

## Introduction

Hypoparathyroidism (HypoPT) is a disease characterized by hypocalcemia and hyperphosphatemia resulting from the absence or inappropriately low levels of PTH. The most common cause is dysfunction of the remaining parathyroid tissue after thyroid or parathyroid surgery.^(^ ^1^ ^)^ Nonsurgical HypoPT, also referred to as idiopathic HypoPT, is less common and mainly a result of autoimmune or genetic disorders.^(^ ^2^, ^3^ ^)^

In contrast with other endocrine disorders, the standard therapy cannot replace the deficient hormone, supplementing active vitamin D and calcium instead. The current method does not restore the physiologic status and is hampered by the occurrence of potentially serious complications, such as renal and basal ganglia calcifications, cataracts, and premature chronic kidney dysfunction.^(^ ^4^, ^5^, ^6^ ^)^ Previous studies on disease characterization using conventional treatment also revealed an increased risk of hospitalization as a result of infection, neuropsychiatric disease,^(^ ^7^ ^)^ abnormal bone architecture,^(^ ^8^, ^9^, ^10^ ^)^ and impaired muscle function.^(^ ^11^ ^)^ Lately, an increase in mortality has also been found.^(^ ^6^ ^)^ Quality of life (QoL) has also been reduced in patients with HypoPT compared with both the normal population and patients undergoing thyroid surgery.^(^ ^2^, ^11^, ^12^, ^13^ ^)^

Recently, the recombinant human parathyroid hormone 1–84 (rhPTH[1–84]) was approved for use in the United States and Europe. Given the lack of long‐term data relating to rhPTH therapy, it is not evident as to whether this therapy will reduce the long‐term complications of HypoPT.

One of the main problems evident in daily patient care is that the principle of cause and effect in such clinical manifestations is unknown. The European guidelines^(^ ^14^ ^)^ and the recommendations developed at the first International Conference on the Management of Hypoparathyroidism, Florence, Italy, May 7–9, 2015^(^ ^15^ ^)^ are an attempt to standardize treatment internationally with the aim of reducing complications and symptoms. Until recently, the defined level of control was based mainly on achieving the target range for serum calcium levels. This has now changed to include multiple biochemical parameters beyond serum calcium, including serum phosphate, calcium–phosphate product (CPP), magnesium, and urinary calcium excretion.^(^ ^14^, ^15^ ^)^

However, outcomes reported by patients with chronic disease are receiving increasing attention. The well‐being of patients is supposed to be a hallmark of therapy. Hence, optimal control of HypoPT should not be restricted simply to restoring biochemical markers. Symptoms of HypoPT are diverse and frequently hard to classify.^(^ ^16^, ^17^ ^)^ It is thus essential to establish and validate tools that help to define optimal disease control.^(^ ^18^ ^)^ This becomes even more important when considering that new methods of treatment, such as rhPTH, are expensive and place an additional burden on the health care system.

Several instruments to assess clinical symptoms and QoL have been used to date. In addition to the QoL questionnaire most commonly applied, the Short Form‐36 (SF‐36), other instruments such as the Hospital Anxiety and Depression Scale (HADS) and the WHO‐5 Well‐Being Index survey (WHO‐5) have also been implemented.^(^ ^2^, ^11^, ^13^, ^19^, ^20^ ^)^ These different questionnaires revealed large deviations from the norms observed in the general population.^(^ ^2^, ^11^, ^19^ ^)^ However, studies involving patient‐control groups instead of population norms have been disappointing when attempting to quantify objectively symptoms subjectively perceived by patients with HypoPT.^(^ ^12^, ^17^ ^)^ Furthermore, the generic SF‐36 tool has proven unable to detect any difference in the QoL in patients treated with rhPTH[1–84] when compared with a control group treated with placebo.^(^ ^17^, ^20^ ^)^

The necessity to better characterize and quantify the nature of the impairment in patients with HypoPT, as well as its relationship to aspects of the disease has been stated a number of times in the literature recently.^(^ ^14^, ^18^, ^20^, ^21^, ^22^ ^)^ The importance of a HypoPT‐specific instrument to assess symptoms is regarded as crucial to improving our understanding of the nature and the degree of impairments to QoL, individual differences, and their relationship to biochemical variables (if any) and treatment modalities.^(^ ^18^, ^20^ ^)^

We recently developed the disease‐specific Hypoparathyroid Patient Questionnaire (HPQ 40/28) to be used in the daily care of patients with HypoPT.^(^ ^23^ ^)^ In the present study, we tested the HPQ 40/28 for 1 year in two endocrine centers in Germany on patients with postsurgical HypoPT as compared with patients who also underwent surgery to treat thyroid disease.

We used a second control group comprised of patients with primary hyperparathyroidism (pHPT) as additional control of disturbances in PTH levels. Medical history, current medication, and biochemical parameters were documented systematically, and we assessed the influence on detected complaints.

## Patients and Methods

### Study design

We conducted a cross‐sectional study in two different endocrinologic centers (Goettingen and Saarbruecken) in Germany from January until December 2016. All HypoPT patients eligible for inclusion in the study in either of the two centers during the recruitment period January to December 2016 were included. We estimated recruiting 40 to 50 HypoPT patients in that period, and aimed to recruit at least twice the number of patients as there are questions on the questionnaire. With the inclusion of control groups of the same size, we considered this possible. In Gottingen, with respect to all three groups, we informed 137 patients selected for potential suitability based on their medical histories, of whom 122 patients agreed to participate. In addition, patients not subject to this preselection were recruited on their first visit to the center.

We were able to recruit 65 patients in the HypoPT group. In the control groups, 49 patients following thyroid surgery without HypoPT (ThySu) and 37 patients with pHPT were recruited. Following age‐ and gender‐matching, 123 patients remained for analysis. Taken together, we included 49 HypoPT patients and 74 control patients (two control groups consisting of 39 ThySu and 35 pHPT patients). Patients were prospectively enrolled in the study based on their clinic attendance. Patients received information on the study during their regular clinical check‐up visits. After providing informed consent, they completed the HPQ 40/28^(^ ^23^ ^)^ form during their visit to the center, and the documentation form was filled in by the attending physician. Biochemical parameters were analyzed as part of the routine control visit, including serum and urinary parameters. For the ThySu group, 24‐hour urine collection was added to the routine work‐up of the patients.

The study was approved by the Ethics Review Board of the University Medical Center Goettingen (No. 25/10/15); all subjects provided written informed consent prior to participation. Data were pseudonymized. All the questionnaires were in German.

### Patients

Patients and controls deemed eligible were identified based on their medical records prior to study onset. Furthermore, patients and controls presenting to the center for the first time could be included after providing informed consent if they fulfilled the inclusion criteria. Chronic postsurgical HypoPT was defined by the presence of hypocalcemia and inappropriately low PTH levels, and patients needing treatment at least 6 months after surgery. The duration of disease in HypoPT patients was 12.55 ± 9.76 years.

HypoPT patients were excluded if their HypoPT proved to be idiopathic, genetic, or transient (less than 6 months diagnosed with HypoPT), they were under 18 or over 85 years of age, pregnant, unable to understand and answer the questionnaires, or were suffering from polyglandular autoimmune syndrome.

Patients with pHPT were either still suffering from or had recently undergone surgery for that condition to consider both the consequences of high PTH levels and the impact of parathyroid surgery. Concomitant secondary pHPT was accepted, whereas no patients with tertiary or solely secondary pHPT were included.

ThySu patients were excluded from participation if they presented autoimmune polyendocrine syndrome or pHPT. Postoperative HypoPT was an exclusion criterion for both control groups.

Because no significant differences regarding gender and age were found among groups (*p* = 0.517 and *p* = 0.166; see Table 1), all patients were analyzed for further testing and comparison. Table 1 lists the characteristics of the three groups.

**Table 1:** Patient Characteristics: Age, Gender, and Disease‐Specific Medication

	HypoPT	ThySu	pHPT	*p* Value^a^ ^,^ ^b^
	*n* (%)	*n* (%)	*n* (%)	
Patients	49 (40)	39 (32)	35 (28)	
Age	57.33 ± 10.52	55.31 ± 9.96	59.91 ± 10.63	0.166^b^
Gender				
Female	41 (84)	36 (92)	29 (83)	0.517^a^
Male	8 (16)	3 (8)	6 (17)	
Active vitamin D				
No intake	7 (14)	39 (100)	33 (97)	**<0.001** ^**a**^
Calcitriol	14 (29)	0	1 (3)	
Alfacalcidol	21 (43)	0	0	
Dihydrotachysterol	6 (12)	0	0	
Combination of 2 different active vitamin D	1 (2)	0	0	
Combined supplements (native vitamin D and calcium)				
No intake	41 (84)	38 (97)	33 (94)	0.098^a^
Calcimagon/calcilac	6 (13)	0	2 (6)	
Calcimed	1 (2)	1 (3)	0	
Calcigen	1 (2)	0	0	
Native vitamin D supplements				
Cholecalciferol/ergocalciferol	22 (45)	21 (54)	22 (65)	0.215^a^
Calcium supplements				
eg, Calcium carbonate	24 (49)	0	0	**<0.001** ^**a**^
Recombinant human PTH 1–34	2 (4)	1 (3)	0	0.779^a^
Thiazides	7 (14)	0	5 (14)	**0.021** ^**a**^
Magnesium	13 (27)	2 (5)	1 (3)	**0.002** ^**a**^
Thyroid hormone	49 (100)	38 (97)	16 (46)	**<0.001** ^**a**^

HypoPT = Hypoparathyroidism; pHPT = primary hyperparathyroidism; Thysu = thyroid surgery without HypoPT. Significant *p*‐values are in bold face.

a Chi‐squared or Fisher's exact for cross‐table calculation.

b One‐way ANOVA.

### Questionnaires

The HPQ 40/28 is a questionnaire we designed based on previous testing to measure the symptoms and complaints typical of HypoPT patients.^(^ ^23^ ^)^ The version we employed here was the original questionnaire comprising 40 items rated on a four‐step scale from 0 (“not at all”) to 3 (“severely”) as indication of the symptom intensity. The original HPQ 40 was subsequently revised and shortened to a 28‐item questionnaire (the HPQ 28) after completing this study. Twelve items were removed from the HPQ 40 that were determined as not belonging to any of the scales identified in this study or not significantly different. Employing the HPQ 28 in place of the HPQ 40 in this study would have no effect on the results at all because the items included in the analysis remained identical. We retain the name HPQ 40/28 here simply as a means of identifying the background of the form. The HPQ28 can be found in the original German version and as English translation in the supplemental material. The questionnaire was both developed and tested in German. Items were translated into English by one person and then translated back into German by another person to validate the translation. Both translators assessed and clarified differences between the original and back‐translated German versions in wording, which was adjusted accordingly in the English version where necessary. The English version is not yet validated.

The number of items in each corresponding scale remained the same, although the item numbering changed: pain and cramps (PaC), including five items (items 3, 6, 12, 14, and 20); loss of vitality (Vit), including six items (items 23 to 28); depression and anxiety (DaA), including 5 items (items 7, 9, 13, 15, and 18); neurovegetative symptoms (NVS), including five items (items 4, 11, 16, 17, and 19); and gastrointestinal symptoms (GIS), including two items (items 8 and 10). Two additional items (items 21, 22) were taken from the Patient Health Questionnaire 2 (PHQ‐2) as an established screening tool for depression.^(^ ^24^, ^25^ ^)^ Three items were not attributable to any of the five scales (items 1, 2, and 5).

### Statistics

Data were analyzed with IBM‐SPSS software versions 22 and 24 (SPSS, Inc, Chicago, IL, USA). Descriptive data were compared using chi‐squared or Fisher's exact test for categorical variables. Answers to items on the HPQ 40/28 were coded as following: 0 = not at all, 1 = slightly, 2 = moderately, 3 = severely. Exploratory factor analysis revealed five scales,^(^ ^23^ ^)^ which were analyzed by calculating the mean value of all items belonging to the corresponding scale. Group differences were evaluated using either ANOVA for normally distributed continuous values or the nonparametric Kruskal–Wallis test (with included Dunn–Bonferroni test in SPSS) for not normally distributed data. With three groups of the sizes 49, 39, and 35 and assuming a within‐group SD using the HPQ 40/28 of 0.7, a difference of 33% from 1.2 in the HypoPT to 0.8 in the control groups was detectable with approximately 80% power using one‐way ANOVA. Post hoc, we applied Bonferroni multiple comparison tests with HypoPT as a reference group to compare all groups. Data are presented as means ± SD or SEM of all patients of the corresponding group for each of the five scales or single items. Correlation between parameters was assessed using Spearman's rho correlation. Receiver operating characteristic (ROC) analysis was applied to compare those hypoPT patients with high symptom load with those patients with fewer symptoms. To separate the two groups, we used the median of the sum of those four scales that were significantly different between the hypoPT and the control groups (Scales: PaC and NVS and the items “numbness or tingling” [item 1] and “heart palpitations” [item 2]; median PaC + NVS + item 1 + item 2 hypoPT group = 5.088). With the hereby identified Youden index, we determined a threshold value. The probability of depression was evaluated by summing up values from the two screening items, whereby a score ≥3 indicated the presence of depression.^(^ ^25^ ^)^

Alpha level was set at *p* = 0.05 and Bonferroni correction for multiple testing was applied depending on the character of the analysis.

## Results

### Patient characteristics

The characteristics of the patients participating in this trial are given in Table 1. There was no significant difference among the three groups with respect to age and gender. The duration of disease in HypoPT patients was 12.55 ± 9.76 years. Patients with pHPT were either still suffering (46%, *n* = 18) or had recently undergone surgery for that condition (54%, *n* = 19). Patients were included in this study after a mean duration of 4.05 ± 4.25 years after surgery (range 1 to 19 years). However, patients presenting with postsurgical HypoPT were excluded from this group.

It is also worth noting that the members of all study groups were predominantly female.

### Medication

The medication administered to participants is given in Table 1. Calcium intake in any tablet form (single or combined with native vitamin D3) was reported by 65.7% of the HypoPT patients. Active vitamin‐D compounds were taken by 85.7%. A combination of calcium (single or combined preparation with native vitamin D3) and active vitamin D was used by 57.1% of patients. Alfacalcidol was the active vitamin‐D preparation most often taken (42.9%), followed by calcitriol (28.6%) and dihydrotachysterol (12.2%). Two patients with HypoPT were treated for HypoPT with rhPTH 1–34 (teriparatide); one patient in the ThySu group was treated for osteoporosis with rhPTH 1–34.

### Extent of thyroid surgery

The underlying disease and type of thyroid surgery in the HypoPT and the ThySu groups are given in Table 2. Patients with HypoPT had undergone surgery for carcinoma and pHPT more often. Of the HypoPT patients, 84% had undergone total thyroidectomy. In contrast, only 56.4% of the ThySu group no longer had their thyroid gland. The other surgical procedures were not followed by a clear increased risk of developing HypoPT in our patients. Our results indicate a significantly higher risk of developing permanent HypoPT after total thyroidectomy, as has been described earlier.^(^ ^26^ ^)^

**Table 2:** Type and Underlying Disease Leading to Thyroid Surgery in the Two Study Groups

	HypoPT	ThySu	Total	*p* Value^a^
	*n* (%)	*n* (%)	*n* (%)	
Type of thyroid surgery				
Total	41 (84)	22 (56)	63 (72)	**0.008**
Hemi	0	7 (18)	7 (8)	
Subtotal	4(8)	4 (10)	8 (9)	
Near total	3 (6)	5 (10)	8 (9)	
Hemi and subtotal	1 (2)	0	1 (1)	
Underlying disease requiring thyroid surgery				
Goiter	21 (43)	18 (47)	39 (45)	0.331
Carcinoma	14 (29)	7 (18)	21 (24)	
Grave disease	6 (12)	5 (13)	11 (13)	
Nodules	3 (6)	7 (18)	10 (12)	
pHPT	2 (4)	0	2 (2)	
Other	3 (6)	1 (3)	4 (4)	

HypoPT = Hypoparathyroidism; ThySu = thyroid surgery without HypoPT.

a Fisher's exact for cross table calculation.

### Biochemical parameters

**Figure 1:**
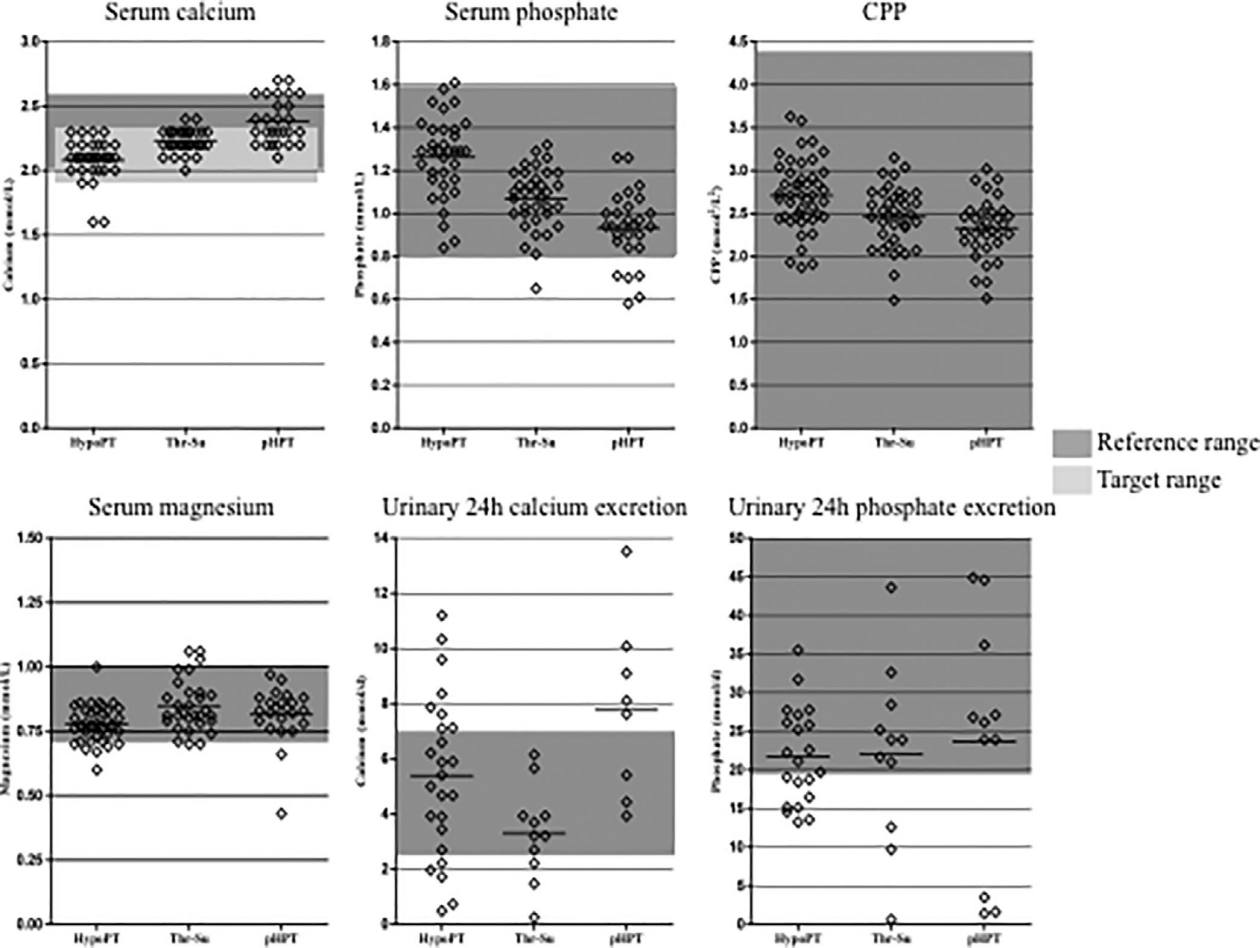
Dot plot and mean values of biochemical parameters in the three study groups corresponding to values in Table 3. CPP = Calcium‐phosphate product; HypoPT = hypoparathyroidism; pHPT = primary hyperparathyroidism; ThySu = thyroid surgery without hypoPT.

To characterize the patient group more effectively, laboratory results for calcium (corrected for albumin), phosphate, CPP, magnesium, phosphate, and urinary calcium excretion over 24 hours are portrayed in Fig. 1 and in Table 3. In the HypoPT patients, 73.9% were found to be within the reference range for calcium (2.0 to 2.6 mmol/L), with 87% lying within the target range (defined as 1.9 to 2.25 mmol/L). In the case of serum phosphate and CPP, 95.7% and 100% of the patients, respectively, were determined as lying within the reference ranges. Determination of 24‐hour calcium excretion in urine revealed 56% of the patients within the reference range, with 20% below and 24% above it. The corresponding percentage of patients within the reference range for urinary phosphate over 24 hours (data not shown) was determined as 57.1%. Urinary phosphate was found to lie below the reference range in 42.9% of patients, but no patients demonstrated values above the reference range.

**Table 3:** Biochemical Parameters in the Three Study Groups

Parameter (reference values)	HypoPT	ThySu	pHPT	All groups	*p* Value^a^
	Mean ± SD	Mean ± SD	Mean ± SD		
Serum calcium (corr.) (2.0 to 2.6 mmol/L)	2.09 ± 0.16 (*n* = 46)	2.23 ± 0.09 (*n* = 35)	2.41 ± 0.17 (*n* = 32)	2.22 ± 0.20 (*n* = 113)	**<0.001**
Serum phosphate (0.8 to 1.6 mmol/L)	1.26 ± 0.19 (*n* = 48)	1.10 ± 0.14 (*n* = 38)	0.93 ± 0.16 (*n* = 33)	1.11 ± 0.22 (*n* = 119)	**<0.001**
CPP (<4.4 mmol^2^/L^2^)	2.71 ± 0.40 (*n* = 46)	2.46 ± 0.25 (*n* = 38)	2.33 ± 0.35 (*n* = 33)	2.52 ± 0.4 (*n* = 117)	**<0.001**
25OHD3 (72.5 to 139 nmol/L)	101.8 ± 46.8 (*n* = 48)	78.3 ± 29.2 (*n* = 38)	86.5 ± 28.7 (*n* = 33)	90 ± 38.3 (*n* = 119)	0.058
Serum magnesium (0.66 to 1.07 mmol/L)	0.78 ± 0.07 (*n* = 40)	0.85 ± 0.08 (*n* = 30)	0.82 ± 0.11 (*n* = 23)	0.80 ± 0.13 (*n* = 94)	0.008
TSH (0.27 to 4.2 mLU/L)	1.11 ± 1.26 (*n* = 32)	1.32 ± 1.61 (*n* = 35)	1.45 ± 0.64 (*n* = 29)	1.29 ± 1.26 (*n* = 96)	0.014
GFR (95 to 110 mL/min/1.73 m^2^)	80.6 ± 17.6 (*n* = 49)	90.6 ± 14.3 (*n* = 39)	85.2 ± 15.9 (*n* = 33)	85 ± 16.6 (*n* = 121)	0.016
Urinary 24‐hour calcium excretion (2.5 to 7.5 mmol/d)	5.39 ± 2.90 (*n* = 25)	3.31 ± 1.70 (*n* = 11)	7.78 ± 3.20 (*n* = 8)	5.30 ± 3.03 (*n* = 44)	0.007
Urinary 24‐hour phosphate excretion (19.37 to 50.5 mmol/d)	21.76 ± 6.23 (*n* = 21)	22.10 ± 11.55 (*n* = 11)	23.63 ± 15.68 (*n* = 11)	22.33 ± 10.46 (*n* = 43)	0.655

GFR = Granular filtration rate; HypoPT = hypoparathyroidism; pHPT = primary hyperparathyroidism; Thysu = thyroid surgery without HypoPT; TSH = thyroid stimulating hormone.

a Kruskal‐Wallis test. Bonferroni correction for the number of parameters (*n* = 9; α ≤ 0.0056), GFR as estimated by Chronic Kidney Disease Epidemiology Collaboration (CKD‐EPI) equation. Values significant after Bonferroni correction are in bold face.

As expected, significant group differences were found for albumin‐corrected serum calcium (*p* < 0.001) and phosphate (*p* < 0.001) in comparison with both control groups (Table 3). CPP was highest in HypoPT patients and significantly different when compared with pHPT patients (*p* < 0.001), owing to the low phosphate level expected for the latter disease.

No other parameters revealed any difference after Bonferroni correction.

Renal function in HypoPT patients is of major interest. At 80.6 mL/min, the glomerular filtration rate (GFR) as estimated by the Chronic Kidney Disease Epidemiology Collaboration (CKD‐EPI) equation^(^ ^27^ ^)^ was lower than both control groups (Table 3). The number of patients in the HypoPT group with a GFR ≤60 mL/min was 12.2%. Thyroid stimulating hormone (TSH) levels did not differ between the groups after Bonferroni correction; however, the number of thyroxine treatments in the group with pHPT was much lower (45.7% of patients in the pHPT versus 100% and 97.4% in the HypoPT and the ThySu, respectively; Table 1). In the case of urinary calcium excretion, both increased and decreased values were detected in HypoPT patients, in contrast to the other two groups corresponding to the underlying disease and its treatment. In the ThySu group, only normal or low values were measured. In the group with hyperparathyroidism—corresponding to the disease—normal or increased values were found.

### HPQ 40/28

The HPQ 40/28 is an instrument we designed to measure the symptoms and complaints of HypoPT patients based on previous testing in self‐help groups.^(^ ^23^ ^)^

**Figure 2:**
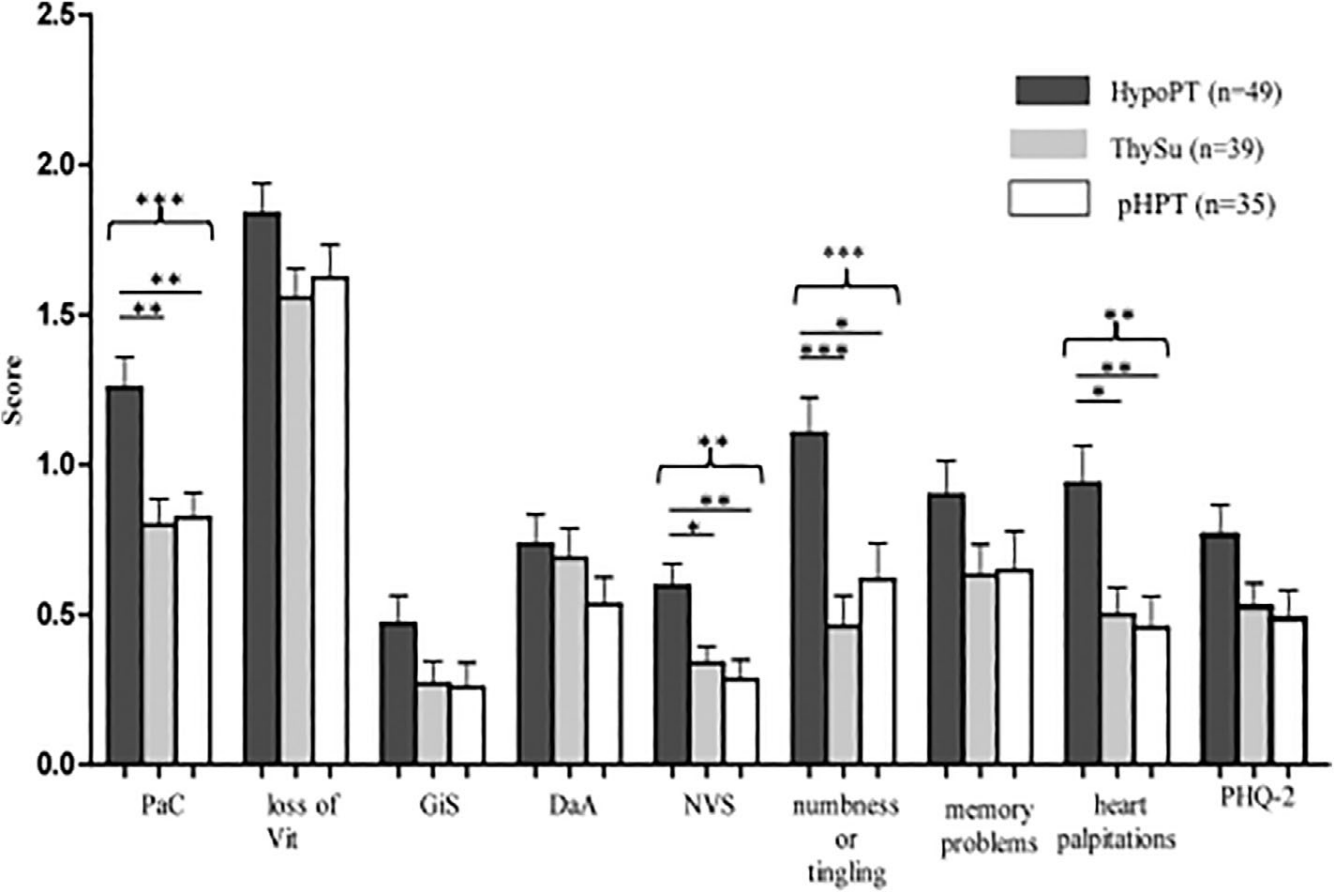
Scores of scales and single items of the HPQ 40/28 comparing HypoPT patients with the two nonhealthy control groups (mean ± SEM). ANOVA followed by Bonferroni comparison testing. ***p < 0.001, **p < 0.01, *p < 0.05. PaC = Pain and cramps; Vit = loss of vitality (here the scale is named loss of vitality for better understanding because higher values indicate less vitality and lower values indicate high vitality); GiS = gastrointestinal symptoms; DaA = depression and anxiety; NVS = neurovegetative symptoms; PHQ‐2 = Patient Health Questiomaire‐2.^(^ ^23^ ^)^

We analyzed the HPQ 40/28 subscales, which revealed significantly higher scores for HypoPT (1.26 ± 0.72) on the PaC subscale compared with both control groups (ThySu: 0.80 ± 0.54 versus pHPT: 0.82 ± 0.50, *p* = 0.001) and on the NVS scale (HypoPT: 0.60 ± 0.52) in comparison with pHPT (0.28 ± 0.39, *p* = 0.002) (Fig. 2). In addition, two single items (“numbness or tingling” and “heart palpitations”) were significantly different from both control groups.

There was no significant difference in the Vit scale in HypoPT (1.84 ± 0.70) compared with the two control groups (ThySu: 1.56 ± 0.61 versus pHPT 1.62 ± 0.67, *p* = 0.121) (Fig. 2).

The scales Vit (*p* = 0.1), GIS (*p* = 0.1), and DaA (*p* = 0.3); the item “memory problems” (*p* = 0.2), as well as the PHQ‐2 depression scale (*p* = 0.1) did not differ significantly between the three study groups (Fig. 2). In accordance with the findings on the “depression and anxiety” scale, the depression screening questions (PHQ‐2) revealed no significant differences between groups (*p* = 0.167). However, 22.4% of the HypoPT patients and only 8.1% and 11.8% of the ThySu and pHPT groups were positive on screening, suggesting a higher number of patients suffering from depression in the HypoPT group.

### Association of laboratory values on scales and items in the HPQ 40/28

It was of main interest as to whether parameters such as laboratory values are associated with these new symptom scales and items as measured by the HPQ 40/28. To analyze the influence of different biochemical parameters, we concentrated on the HypoPT group exclusively (Table 4).

**Table 4:** Correlation of Biochemical Parameters to the Scales and Items of the HPQ 40/28 in the HypoPT Study Group

Parameter	Spearman rhocorrelation	PaC	Vit	GiS	DaA	NVS	Numbness or tingling	Memory problems	Heart palpitations
Serum calcium (mmol/L)	*r*_s_ ^a^	0.285	0.175	0.265	0.252	**0.309**	0.181	0.262	0.249
	*p* Value^b^	0.055	0.246	0.075	0.091	**0.037**	0.229	0.079	0.099
	*n* ^c^	46	46	46	46	46	46	46	45
Serum phosphate (mmol/L)	*r*_s_	0.095	**0.349**	−0.063	0.111	−0.003	0.194	0.066	0.147
	*p* Value	0.520	**0.015**	0.670	0.453	0.983	0.192	0.656	0.323
	*n*	48	48	48	48	48	47	48	47
Serum magnesium (mmol/L)	*r*_s_	0.122	−0.019	−0.046	0.035	0.282	0.145	0.218	0.003
	*p* Value	0.454	0.910	0.778	0.831	0.078	0.380	0.176	0.986
	*n*	40	40	40	40	40	39	40	39
CPP (mmol^2^/L^2^)	*r*_s_	**0.294**	**0.498**	−0.015	0.267	0.172	**0.328**	**0.296**	0.228
	*p* Value	**0.047**	**0.0004**	0.923	0.073	0.253	**0.026**	**0.046**	0.132
	*n*	46	46	46	46	46	46	46	45

Because of the exploratory nature of the analysis, Bonferroni correction was not applied. With Bonferroni correction, Vit remained significant.

DaA = Depression and anxiety; GiS = gastrointestinal symptoms; HPQ = Hypoparathyroid Patient Questionnaire; HypoPT = hyperparathyroidism; NVS = neurovegetative symptoms; PaC = pain and cramps; Vit = loss of vitality (the scale is named loss of vitality for better understanding because higher values indicate less vitality and lower values indicate greater vitality).

a *r*_s_ = coefficient parameter (Spearman rho correlation).

b Significance for Spearman rho correlation for the null hypothesis was true.

c *n* = number of analyzed values.

With reference to laboratory parameters in the group of HypoPT patients, higher values of serum calcium correlated to NVS.

Serum phosphate levels correlated to the Vit scale, linking higher phosphate levels with a loss of vitality (*r*_s_ = 0.349, *p* < 0.05; Table 4). This result was different from the effect of serum calcium values. Beside these serum parameters, CPP also influenced scales as demonstrated in Table 4. A higher CPP increased the scale for PaC significantly, with *r*_s_ = 0.294. In addition, patients with higher values for the item numbness or tingling also demonstrated a higher CPP.

Interestingly, although not part of the significantly different scales compared with the two control groups, a higher CPP also correlated to memory problems (*r*_s_ = 0.296, *p* = 0.046). The Vit scale, itself revealing no differences between the three study groups, correlated strongly and significantly with CPP (*r*_s_ 0.498, *p* < 0.001). This shows a reduction in QoL as the CPP value increases. This might be explained, in part, by higher serum phosphate values. No other laboratory parameter correlated to the different symptom scales.

### Receiver operating characteristics (ROC) for calcium–phosphate product

**Figure 3:**
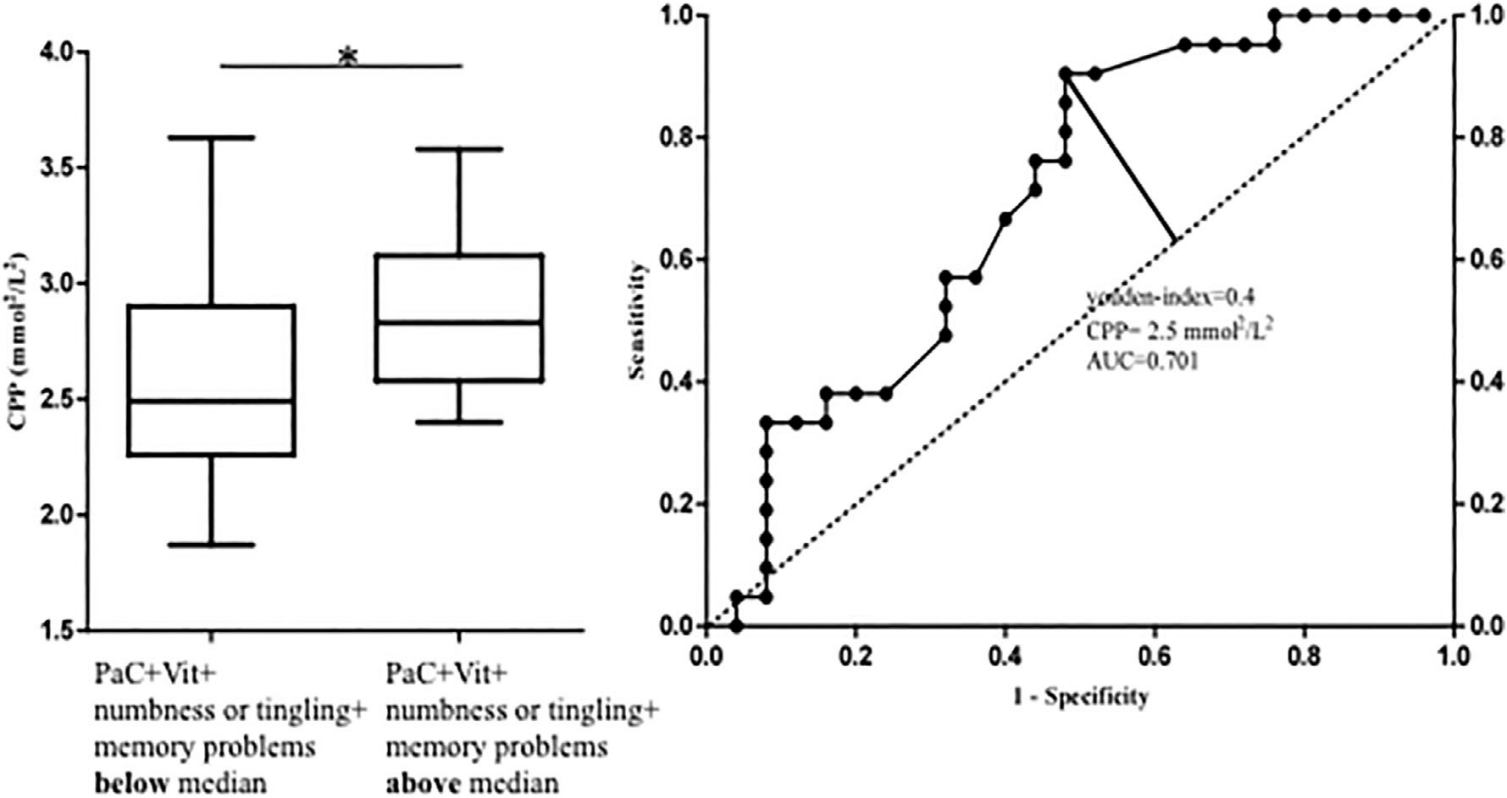
(*A*) Dichotomous determining criterion (above or below median of the scales PaC, Vit, numbness or tingling, and memory problems) and (*B*) resulting ROC curve illustrating the identified Youden index and area under‐the‐curve (AUC) values. CPP = Calcium‐phosphate product; PaC = pain and cramps; Vit = loss of vitality.

Given the high relevance of CPP for two different scales (PaC and Vit) and two single items (numbness or tingling and memory problems), it was of great interest to us to identify a possible threshold value under which the complaints would significantly decrease. We therefore created a receiver operating characteristics (ROC) curve including the two significant scales (PaC, Vit) and the two significant items (numbness or tingling, memory problems). Figure 3*A* represents the dichotomous criterion below and above the sum of the medians of the four parameters. This selection procedure resulted in the ROC curve we present in Fig. 3*B*. The value of 0.7 was determined as the area under the curve (AUC), demonstrating a classification for CPP between fair (AUC 0.7 to 0.8) and poor (AUC 0.6 to 0.7).^(^ ^28^ ^)^ We determined a threshold value for CPP using the Youden index. From this analysis, the threshold value to differentiate between patients with fewer or more complaints lays around 2.5 mmol^2^/L^2^. Using this value, we reached a sensitivity of 0.9, that is, we detected 90% of those with high symptom load. The specificity we reached was 0.5; this implies that 50% of those with low symptom load are above this value of 2.5 mmol^2^/L^2^.

## Discussion

This 1‐year cross‐sectional study aimed to validate the use of the HPQ 40/28 as a specific questionnaire to explore the symptoms and complaints of HypoPT patients. We examined 123 patients, including 49 with postsurgical HypoPT, 39 ThySu patients, and 35 patients with pHPT. The HPQ 40/28 detected specific complaints of HypoPT patients compared with these two corresponding control groups.

**Figure 4:**
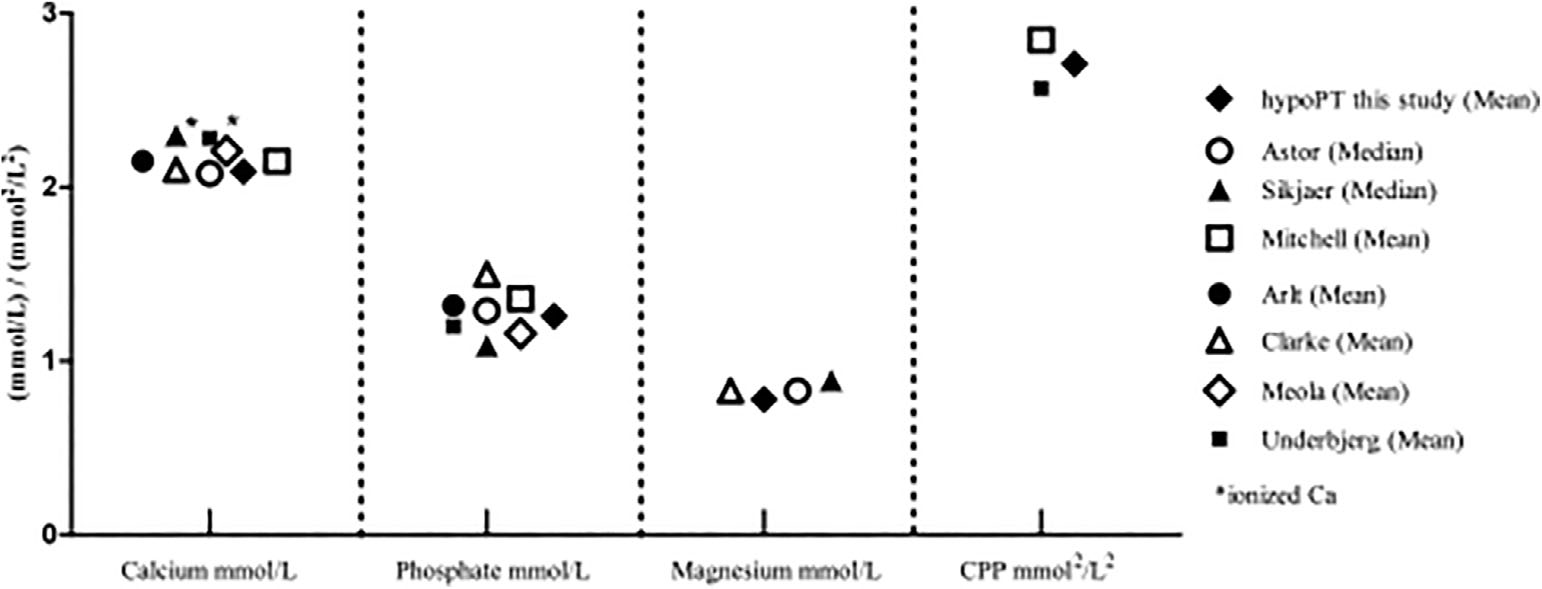
Comparison of published biochemical parameters in different study populations.^(^ ^2^, ^4^, ^11^, ^12^, ^20^ ^)^ CPP = Calcium‐phosphate product; HypoPT = hypoparathyroidism.

The HPQ 40/28 instrument was originally developed comparing HypoPT patients with population norms by using the most relevant or significantly different items.^(^ ^23^ ^)^ These items, represented by the HPQ 40/28, have now been tested in comparison with two corresponding control groups. Although biochemical values in our HypoPT group were within the target range defined by the European guidelines,^(^ ^14^ ^)^ in 87% to 100% of participants, patients reported a number of complaints via the questionnaire. The biochemical parameters in our patients were not different from those of other study populations (Fig. 4).^(^ ^2^, ^4^, ^11^, ^12^, ^29^, ^30^ ^)^ Therefore, the HPQ 40/28 might be judged as appropriate to detect corresponding symptoms in other investigated HypoPT populations.

One unique aspect of value in this study is the inclusion of the two age‐matched control groups with a disease correlating to HypoPT patients. We found that the surgical procedure performed had no influence on the HPQ 40/28 results (data not shown). Both of these groups underwent surgery and were administered thyroid medication.

The ThySu control group seemed very relevant with respect to the results on depression. We discovered no significant differences between HypoPT and ThySu with regard to depression and anxiety. This suggests that the thyroid medication administered or even the surgery itself plays a role in the development of depression. Only one other research group has investigated women (25 women) with HypoPT: they were compared with age‐matched female controls who were operated on for thyroid disease without HypoPT. Their results revealed an increased physical complaint score, but otherwise only tendencies in differences using conventional questionnaires.^(^ ^12^ ^)^ In addition to the group undergoing thyroid disease surgery, we integrated a control group with patients presenting high levels of PTH. This group was particularly relevant, as it controls for PTH as a hormone per se. In addition, this group included 54% surgery patients with cured pHPT. Thus, the pHPT control group is also a strong control for unspecific symptoms as a consequence of surgery not resulting in HypoPT or thyroid dysfunction.

In our study, pain as a relevant symptom was represented in the subscale of PaC in patients with HypoPT when compared with both control groups. The predominant complaints were muscle PaC, as well as bone and joint pain.^(^ ^23^ ^)^ Pain as a complaint was also documented in other populations presenting HypoPT, with up to 51% of patients suffering from pain and muscle cramps.^(^ ^16^ ^)^ In an online study by Hadker and colleagues, three of the six most physical symptoms were muscle PaC, bone and joint pain, and heaviness and weakness in extremities.^(^ ^31^ ^)^ In addition, two German studies using questionnaires detected pain as a symptom. Arlt and colleagues suggested that low magnesium intake or low serum magnesium levels were causative.^(^ ^12^, ^32^ ^)^ Given our finding that magnesium levels did not correlate with the reported pain, it is unlikely that pain is solely caused by hypomagnesemia.^(^ ^16^ ^)^

Hot flushes, chills, dizziness, and diarrhea^(^ ^23^ ^)^ were statistically closely related symptoms reported by a significant number of our patients with HypoPT compared with the patient control groups. Until now, these symptoms have not been described as characteristic of HypoPT.

Steatorrhea caused by a deficit in exocrine pancreas secretion has been mentioned as a major digestive manifestation of HypoPT.^(^ ^33^ ^)^ In another study, 46% of patients experienced disturbance of bowel movements, 37% cold sensations, and 44% heat intolerance as described symptoms.^(^ ^31^ ^)^ Diarrhea could also be indicative of side‐effects to calcium or magnesium treatment. The above described symptoms were identified by our analysis as specific symptoms of patients with hypoPT and are included in the scale neurovegatative symptoms (NVS).

In addition to these two scales, we identified two single items as significantly different between HypoPT patients and control groups. Numbness or tingling are well‐known symptoms of low calcium levels and are described as symptoms in all other populations.^(^ ^2^, ^11^, ^13^, ^31^ ^)^ However, until now they have not been included in any specific questionnaire and thus have not been measureable. Given our results, we deem it appropriate to include these manifestations of disease in assessing disease‐related symptoms.

The HPQ 40/28 identified heart palpitations or a racing heart as relevant complaints. Cardiac effects in HypoPT patients are mainly caused by disease‐specific hypocalcemia according to current knowledge. The main finding on an ECG recorded for these patients is QT‐elongation, which can cause life‐threatening arrhythmias. Furthermore, low serum calcium levels reduce cardiac contractility.^(^ ^34^, ^35^ ^)^ In addition, PTH itself has a direct effect on cardiomyocytes by binding to the PTH1 receptor and affecting calcium influx into the cells and thereby their contractility.^(^ ^36^ ^)^ Heart complaints and palpitations were also detected in a study using different questionnaires. However, the authors interpreted these symptoms as physical equivalents of the psychiatric aspects of HypoPT disease on the grounds of high scores for anxiety, including phobic anxiety.^(^ ^12^ ^)^ Recent data on cardiac contractility and the direct effects of PTH on cardiomyocytes may suggest that cardiac arrhythmias precede the anxiety perceived and therefore need to be regarded as causal and not consequential to anxiety in patients with HypoPT. We conclude that the HPQ 40/28 reflects symptoms that might be correlated to cardiac involvement in HypoPT patients.

Some of the scales of the HPQ 40/28 demonstrated higher values in the HypoPT group, but without reaching significance. This might result from the greater number and intensity of complaints of the two control groups compared with the normative control used to create the new questions in the HPQ40/28. For the different, nonsignificant scales, a greater number of patients will perhaps reveal whether we will be able to capture and quantify these complaints in the future.

To better understand the clinical relevance of the identified scales, we investigated influencing parameters.

The CPP was identified as the most‐interesting biochemical parameter. This parameter correlated with four scales influencing PaC, Vit, numbness or tingling, and memory problems. When only serum calcium was analyzed, an increase in neurovegetative complaints correlated to higher serum levels. This finding was unexpected because clinical experience associates neurovegetative complaints with low calcium levels. However, the questions included in the NVS score refer to trembling muscles, hot flushes or the chills, weakness, dizziness or a feeling of onset syncope, and diarrhea. Typical signs of low calcium levels, such as numbness or tingling, are referred to in separate scales and are not in the NVS scale. Furthermore, the symptom, muscle cramps, attributed to the statistics of the PaC scale, is also associated with low calcium levels. Therefore, the symptoms included in the NVS scale seem to be more sensitive to high calcium levels as suggested by our results.

Lately, HypoPT patients have presented with increased mortality, heart disease, renal disease, and infection rates,^(6)^ with a strong correlation to laboratory parameters. However, studies on QoL have not revealed any correlation with laboratory parameters or medications yet.^(^ ^11^, ^12^ ^)^ This might be because of a lack of symptom‐related specificity on general QoL scales. Normal values for CPP have been established for patients with renal disease. However, no normal or optimal values for HypoPT patients have been published to date.^(^ ^14^ ^)^ A 4.36‐fold increase in mortality rate was observed with a CPP above 2.62 mmol^2^/L^2^ in a recent study by Underbjerg and colleagues.^(^ ^6^ ^)^ In addition, values for the CPP ≥2.93 mmol^2^/L^2^ doubled the risk of any renal disease. Our results also reflect the significance of the CPP value in relation to the complaints of HypoPT patients. We used the ROC approach to analyze the significance of the CPP values and detected a possible threshold value of 2.5 mmol^2^/L^2^. Those hypoPT patients with CPP values below this level demonstrated a significantly lower level of complaints in our study. Perhaps our results in combination with the findings on long‐term complications and mortality^(^ ^6^ ^)^ will help to adapt the CPP reference ranges for patients with HypoPT.

The CPP in combination with serum phosphate levels in HypoPT patients is a crucial laboratory parameter for short‐term complaints, as we have now demonstrated in this study using the HPQ 40/28.

## Strength and Limitations

The strength of the study was the cross‐sectional, two‐center design and the use of two nonhealthy control groups. In addition, we can provide all data on laboratory parameters as suggested by the current guidelines. Furthermore, our study has the advantage that it included patients administered different forms of active vitamin‐D medication. Correlation with laboratory parameters was analyzed as an exploratory approach. After Bonferroni correction, Vit still correlated significantly with the CPP. Hence, we assume that the explorative analysis is correct, but that the correlation to the other parameters has to be confirmed in a greater number of patients.

In summary, we tested a new disease‐specific questionnaire, quantifying symptom categories in patients with HypoPT. This questionnaire successfully identified specific symptoms by which HypoPT patients differed from the two control groups. The quantification permitted us to analyze the associations of laboratory parameters with symptoms’ scales. CPP was identified as the predominant factor in the severity of complaints in HypoPT patients. Our newly developed disease‐specific questionnaire for HypoPT patients and its revised version can now be implemented in further studies and in daily practice with individual patients with special indications.

## Disclosures

During the last 3 years, CH‐L has received lecture honoraria from Heel, Servier, and Novartis, as well as royalties from Hogrefe Huber publishers. HS has served during the last 3 years as an advisory board member for Shire, UCB, and Kyowa Kirin and received speaker's fees from Shire and Amgen. The other authors state no conflicts of interest.

This research did not receive any specific grant from any funding agency in the public, commercial, or not‐for‐profit sector.

## Supporting information


**Figure S1**: Flow chart

**Appendix S1**: Supporting Information

**Appendix S2**: Supporting Information
