# Supplementary figures and images for: Quantification of Symptom Load by a Disease‐Specific Questionnaire HPQ 28 and Analysis of Associated Biochemical Parameters in Patients With Postsurgical Hypoparathyroidism
Source: JBMR Plus. 2020 Jun 5;4(7):e10368. doi: 10.1002/jbm4.10368 (PMC7340443; doi:10.1002/jbm4.10368)

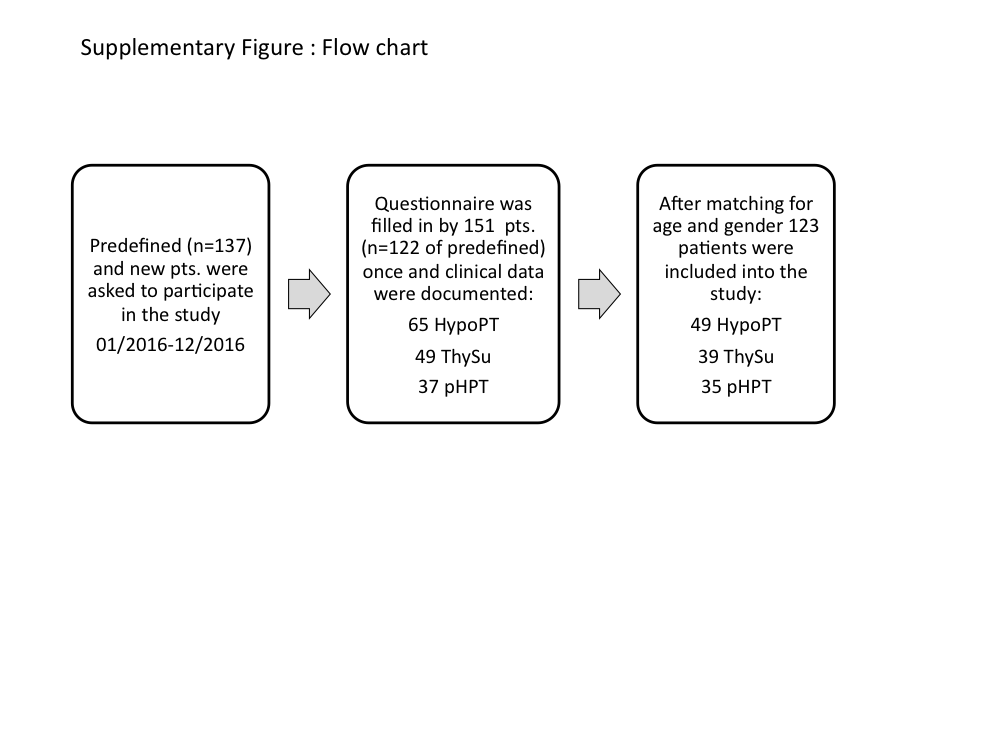

Supplement: Supplementary file 1 — Figure S1: Flow chart [file JBM4-4-e10368-s001.tif]
