# Supplementary material for: Quantification of Symptom Load by a Disease‐Specific Questionnaire HPQ 28 and Analysis of Associated Biochemical Parameters in Patients With Postsurgical Hypoparathyroidism
Source: JBMR Plus. 2020 Jun 5;4(7):e10368. doi: 10.1002/jbm4.10368 (PMC7340443; doi:10.1002/jbm4.10368)
Supplement: Supplementary file 2 — Appendix S1: Supporting Information [file JBM4-4-e10368-s002.PDF]

HPQ 28 - Fragebogen für Hypoparathyreodismus

Name/Nr.:_______________________________________ Datum:_______________________

Dieser Fragebogen enthält verschiedene Fragen zu Symptomen und Beschwerden, die im Rahmen einer Nebenschilddrüsenunterfunktion auftreten können.
Bitte überlegen Sie bei jeder Frage, welche Antwort auf Sie am besten zutrifft und machen Sie ein Kreuz im entsprechenden Feld. Beantworten Sie bitte jede Frage.

Wie sehr litten Sie in den letzten vier Wochen unter… ?	gar nicht	ein wenig	ziemlich	stark
1. …Taubheit oder Kribbeln in bestimmten Körperteilen?	□	□	□	□
2. …Gedächtnisschwierigkeiten?	□	□	□	□
3. …Kreuz- und Rückenschmerzen?	□	□	□	□
4. …Muskelzittern?	□	□	□	□
5. …Herzklopfen oder Herzrasen?	□	□	□	□
6. …Gelenk- oder Gliederschmerzen?	□	□	□	□
7. …Selbstvorwürfen?	□	□	□	□
8. …Übelkeit oder Magenverstimmungen?	□	□	□	□
9. …innerer Anspannung und Unruhe?	□	□	□	□
10. …Bauchschmerzen oder Bauchkrämpfen?	□	□	□	□
11. …Hitzewallungen oder Kälteschauern?	□	□	□	□
12. …Muskelschmerzen?	□	□	□	□
13. …sorgenvollen Gedanken?	□	□	□	□
14. …Nacken- oder Schulterschmerzen?	□	□	□	□
15. …Schwermut?	□	□	□	□
16. …Schwächegefühl?	□	□	□	□
17. …Schwindelgefühlen oder dem Gefühl in Ohnmacht zu fallen?	□	□	□	□
18. …Entscheidungsschwierigkeiten?	□	□	□	□
19. …Durchfall?	□	□	□	□
20. …Muskelkrämpfen?	□	□	□	□

Wie oft haben Sie sich im Verlauf der letzten zwei Wochen beeinträchtigt gefühlt durch… ?	überhaupt nicht	an einzelnen Tagen	an mehr als der Hälfte der Tage	beinahe jeden Tag
21. …weniger Interesse und Freude an Ihren Tätigkeiten?	□	□	□	□
22. …Niedergeschlagenheit, Schwermut oder Hoffnungslosigkeit?	□	□	□	□

Im nächsten Abschnitt stellen wir Ihnen einige Fragen zur Lebensqualität. Die Antwort "gar nicht" bedeutet bei diesen Fragen, dass Sie in Ihrem Wohlbefinden stark eingeschränkt sind. Die Antwort "sehr" bedeutet in diesem Zusammenhang, dass Sie wenige oder kaum Einschränkungen bezüglich Ihres Wohlbefindens haben.

Inwiefern…	gar nicht	ein wenig	ziemlich	sehr
23. …fühlten Sie sich in den letzten vier Wochen energiegeladen?	□	□	□	□
24. …fühlten Sie sich in den letzten vier Wochen körperlich fit und vital?	□	□	□	□
25. …hatten Sie die letzten vier Wochen Freude an Sex	□	□	□	□
26. …waren Sie in den letzten vier Wochen ruhig und ge	□	□	□	□
27. …waren Sie in den letzten vier Wochen glücklich?	□	□	□	□
28. …fühlten Sie sich in den letzten vier Wochen gesund?	□	□	□	□

© 2019 -einige Rechte vorbehalten, siehe "The HPQ—Development and First Administration of a Questionnaire for Hypoparathyroid Patients“, JBMRPlus (WOA), Vol. 4, No. 1, January 2020, e10245,
